# Determination of the median effective dose of sufentanil for inhibiting the laryngeal mask insertion response in geriatric patients: a prospective, double-blinded, dose–response trial

**DOI:** 10.1186/s12871-022-01758-7

**Published:** 2022-07-11

**Authors:** ShiFang Wang, WeiBing Wang, JinBo Xiao, HongPing Yu, Hui Zhou, Huang Xu

**Affiliations:** Department of Anesthesiology, The Affiliated AnQing Municipal Hospitals of Anhui Medical University, 352th, Renming Road, AnQing, 246003 AnHui Province China

**Keywords:** Sufentanil, Etomidate, Laryngeal mask airway, Geriatric, Median effective dose

## Abstract

**Background:**

Laryngeal mask airway(LMA) have been widely used in clinical practice. Irritation to the patient during the insertion of a laryngeal mask can cause hemodynamic fluctuations, which is particularly unsafe for geriatric patients. We used probit regression analysis to determine the median effective dose of sufentanil to inhibit the response to LMA insertion in geriatric patients.

**Methods:**

A total of 90 patients were selected for the study using the following inclusion criteria: age ≥ 65 years old, ASA grade I–III, and scheduled to undergo intravenous general anesthesia with LMA insertion. Each patient received a dose of sufentanil for anesthesia induction in one of six levels: 0.05, 0.1, 0.15, 0.2, 0.25, or 0.3 μg kg^−1^. LMA insertion was scored with a 3-point, 6-category scale, with scores ≥ 16 indicating effective LMA insertion, and < 16 indicating ineffective LMA insertion. Mean arterial blood pressure (MAP), heart rate (HR), and bispectral index (BIS) were recorded 1 min before induction (T1), 1 min after induction (T2), 1 min after LMA insertion (T3), and 5 min after LMA insertion (T4) in each group. In addition, the plasma norepinephrine (NE) levels and adverse reactions were measured at T2 and T3 in each dosage group.

**Results:**

Probit regression analysis showed that the ED50 of sufentanil inhibiting the response to LMA insertion in geriatric patients was 0.18 μg kg^−1^ (95% CI: 0.16–0.21 μg kg^−1^), and the ED95 was 0.31 μg kg^−1^ (95% CI: 0.27–0.38 μg kg^−1^), and the probit(p) = -2.34 + 12.90 × ln(Dose)($\chi^{2}$ = 0.725, *p* = 0.948). Among all the patients, the number of effective LMA insertions was 57 (group A), and the number of ineffective LMA insertions was 33 (group B). The MAP, HR, and NE in group B were significantly higher than in group A at T3.

**Conclusions:**

Sufentanil can effectively inhibit the patient’s response to LMA insertion, with stable hemodynamics and small stress response. The ED50 and ED95 were 0.18 μg kg^−1^ (95% CI: 0.16–0.21 μg kg^−1^) and 0.31 μg kg^−1^(95% CI: 0.27–0.38 μg kg^−1^), respectively.

**Trial registration:**

This study was registered in the Chinese Clinical Trial Registry (Registration number: ChiCTR2100051827) on October 6, 2021.

## Background

According to the latest Chinese census results from 2021, the proportion of the population over the age of 65 has reached 13.5%. With the aging of the population, more and more geriatric patients need surgery. Due to the functional decline of vital organs in geriatric patients, the compensatory response to stress starts slowly and the degree of compensation is small. Therefore, it is important to implement stable anesthesia induction for geriatric patients. The LMA can quickly establish the airway, with little stimulation to the patient, and the cardiovascular response to the insertion is mild [1], with no need for muscle relaxants. The LMA can retain spontaneous breathing, prevent accidental occurrence of mask or intubation difficulties during induction [2], and greatly improve the safety of general anesthesia induction in geriatric patients.

Some non-medical factors also significantly influence the insertional response of the LMA, such as the type of LMA is also an important factor affecting the LMA insertion response. I-gel LMA, a noninflatable cuff made of thermoplastic elastomer, has a successful and shorter duration of insertion, and with less hemodynamic response compared to classic LMA with an inflatable cuff during general anesthesia [3]. Moreover, Supreme LMA requires more lower concentration of end-tidal sevoflurane than ProSeal LMA insertion [4].

When the LMA is inserted, it stimulates the throat and causes discomfort to the patient, so appropriate induction of anesthesia is often required. The preferred anesthetic for LMA insertion is propofol [5], because of its ability to inhibit laryngeal reflexes and relax the jaw [6]. However, propofol has a strong circulatory inhibitory effect, which often causes a significant drop in blood pressure, which is more pronounced in geriatric patients or patients with insufficient effective circulating blood volume. Because etomidate has little effect on circulation [7, 8], it is often used for induction in geriatric patients, but etomidate does not inhibit the upper airway reflex, and it can make LMA insertion very difficult when used alone, so it needs to be combined with opioids. Among the opioids used, sufentanil is often used for anesthesia in the elderly. Compared with fentanyl, sufentanil can relieve myocardial stress in geriatric patients [9], and increase hemodynamic stability and brain tissue oxygenation after anesthesia induction [10].

Although sufentanil has advantages in anesthesia induction in geriatric patients, inappropriate doses can have adverse effects. If the dose is too small, when the LMA is inserted, the patient may experience physical movement, choking, laryngospasm, and severe hemodynamic fluctuations, resulting in failure of the procedure and adverse cardiovascular events; if the dose is too large, cardiovascular function may be inhibited, resulting in a drop in blood pressure and slowing of the heart rate. Therefore, some researchers have studied the optimal dose of sufentanil when the LMA is inserted. Roshdi [11] studied the optimal effect-site concentration of sufentanil when a LMA is inserted under the induction of 4.0 μg mL^−1^ target-controlled infusion of propofol. Li et al. [12] studied the optimal dose of sufentanil for satisfactory LMA intubation in Chinese pediatric patients under the induction of 2.5 mg kg^−1^ propofol. At present, there are few studies on the required dose of sufentanil for LMA insertion in geriatric patients during etomidate induction. Our study explored the median effective dose of sufentanil to inhibit the response to LMA insertion in geriatric patients.

## Methods

### Design

We conducted a prospective, double-blinded, dose–response trial to determine the median effective dose of sufentanil for inhibiting the laryngeal mask insertion response in geriatric patients.

### Subjects and setting

A total of 90 patients who were ≥ 65 years old, had ASA grade I–III, and were scheduled to undergo intravenous general anesthesia with LMA insertion in our hospital were enrolled in the study.This study was approved by the Ethics Committee of Anqing Hospital Affiliated to Anhui Medical University, and all patients signed informed consent. On October 6, 2021, our study was registered at the China Clinical Trial Registration Center (Registration number: ChiCTR2100051827).

Exclusion criteria were as follows: severe hypertension (systolic blood pressure ≥ 180 mmHg or diastolic blood pressure ≥ 110 mmHg), long-term use of opioid analgesics, BMI > 30 kg/m^2^, drug allergy, adrenal insufficiency, or unsuitability for LMA insertion.

### Study protocol

After the patient entered the operating room, we opened the venous channel for infusion, monitored the patient’s blood pressure, electrocardiogram, pulse oxygen saturation (SpO_2_), body temperature, and bispectral index (BIS), intravenously injected midazolam 0.04 mg kg^−1^, put the patient on mask oxygen inhalation at 5 L min^−1^, and performed anesthesia induction after 10 min. Sufentanil (Humanwell Healthcare, Hubei, China) was formulated by the anesthesia nurse into six doses: 0.05, 0.1, 0.15, 0.2, 0.25, and 0.3 μg kg^−1^. Each dose was prepared in 0.9% normal saline to a total of 5 mL and was then given a number. The anesthesia nurse did not participate in subsequent experiments. A random number was generated by the computer for each patient, and the dose matching the generated number was used for the patient. This way, the anesthesiologist did not know the induction dose of each patient. Sufentanil was slowly injected for more than 30 s. After 3 min, 0.3 mg kg^−1^ etomidate was slowly injected for more than 30 s (Nhwa Pharmaceutical, Jiangsu, China). An I-gel LMA was inserted 2 min later, when the patient lost consciousness and the eyelash reflex disappeared. The patient was connected to the anesthesia machine and the waveform of end-tidal carbon dioxide tension was monitored (machine-assisted breathing was performed if there was no spontaneous breathing). If the LMA showed poor ventilation or air leakage, the LMA was re-inserted and tested again. If satisfactory ventilation was not achieved after two attempts, rocuronium was injected for endotracheal intubation. Mechanical ventilation method: Tidal volume 8–10 ml kg^−1^, Ventilation frequency 12 beats min^−1^, and I:E was 1:2, ventilation parameters were adjusted according to PetCO_2_, and PetCO_2_ was maintained between 35 and 45 mmHg. The respiratory depression of the patients in the induction period was observed. If the SpO_2_ was lower than 92%, an oxygen mask was provided to assist breathing. Five minutes after LMA insertion, we intravenously pumped cisatracurium 2–5 μg kg^−1^ min^−1^, propofol 2–6 mg kg^−1^ h^−1^, and remifentanil 0.1–0.2 μg kg^−1^ min^−1^ for intraoperative maintenance; analgesics were given before the operation. If the mean arterial blood pressure (MAP) was 20% lower than the baseline blood pressure, 5–10 mg of ephedrine was intravenously injected. If the heart rate (HR) was lower than 50 beats/min, 0.3–0.5 mg of intravenous atropine was administered.

### Measurements

The following outcomes were assessed: (1) The LMA insertion condition was assessed by the anesthetic assistant, who remained blinded to the dose of sufentanil. The LMA insertion conditions were evaluated through a 3-point, 6-category scale (Table 1); (2) Hemodynamics: MAP, HR, and BIS at 1 min before induction (T1), 1 min after induction (T2), 1 min after LMA insertion (T3), and 5 min after LMA insertion (T4); (3) 2 ml of venous blood was drawn at T2 and T3, and the plasma norepinephrine (NE) concentration was measured by high performance liquid chromatography tandem mass spectrometry (LC–MS/MS); (4) Adverse effects: Ephedrine or atropine required during the induction period, duration of apnea > 2 min, or any of the following adverse reactions: choking, muscle stiffness, injection pain, muscle tremor, intraoperative awareness, or throat discomfort.

**Table 1:** The score of LMA insertion

Behavior	Score
Resistance to mouth opening grading	no = 1, significant = 2, undue force required = 3
Resistance to insertion grading	no = 1, significant = 2, undue force required = 3
Swallowing grading	nil = 1, slight = 3, gross = 3
Coughing and gagging grading	nil = 1, slight = 3, gross = 3
Head or body movement grading	nil = 1, slight = 3, gross = 3
Laryngospasm grading	nil = 1, partial = 2, total = 3

A score of ≥ 16 was defined as “effective” and a score of < 16 defined as “ineffective”

### Statistical analysis

The Cochran-Armitage Test for the trend in the incidence of inhibiting the LMA insertion response was utilized to perform sample size calculations using PASS (version 11.0.7; NCSS, LLC, Kaysville, UT). Based on our preliminary data that incidence of inhibiting the LMA insertion response was 9, 14, 33, 56, 90, and 94%, respectively for the six sufentanil injection doses of 0.05, 0.1, 0.15, 0.2, 0.25 and 0.3 μg kg^−1^, our calculation indicated that a total sample size of 72 patients (12 per group) would provide 82% power to detect a linear trend using a two-sided Z test and a significance level of 0.05. To account for possible dropouts, we enrolled 15 patients for each group, so the 90 patients is an adequate sample size.

All data were analyzed with the SPSS 21.0 statistical software. Normally distributed measurement data were expressed as mean ± standard deviation ($\overline{\mathrm{x} }$ ± SD), and independent sample *t*-test was used for comparison. Categorical data were compared using the χ^2^ test or Fisher's exact probability method. The median effective dose of sufentanil was analyzed by probit regression analysis. *P* < 0.05 was considered statistically significant.

## Results

Demographic characteristics (age, weight, and BMI), ASA classification, and duration of surgery were compared between the two groups (Table 2).

**Table 2:** Demographic characteristics, ASA status, BMI, hypertension, and duration of surgery

	Group A (*n* = 57)	Group B (*n* = 33)	*P*-value
Age (years)	72.6 ± 5.2	72.2 ± 5.5	0.732^△^
Weight (kg)	60.2 ± 9.2	58.9 ± 9.5	0.490^△^
BMI (kg/m^2^)	22.7 ± 2.3	22.5 ± 2.7	0.715^△^
Gender (M/F)	34/23	22/11	0.508^※^
Hypertension (Y/N)	20/37	14/19	0.489^※^
ASA (I/II/III)	2/46/9	1/28/4	0.633^※^
Duration of surgery (min)	66.1 ± 29.9	63.4 ± 29.5	0.684^△^

Data are presented as the mean ± SD or the number

*M* Male, *F* Female, *Y* Yes, *N* No, *BMI* Body mass index, *ASA* American Society of Anesthesiologists

^△^*P*-value: Student t-test

^※^*P*-value: Chi-square test

**Fig. 1:**
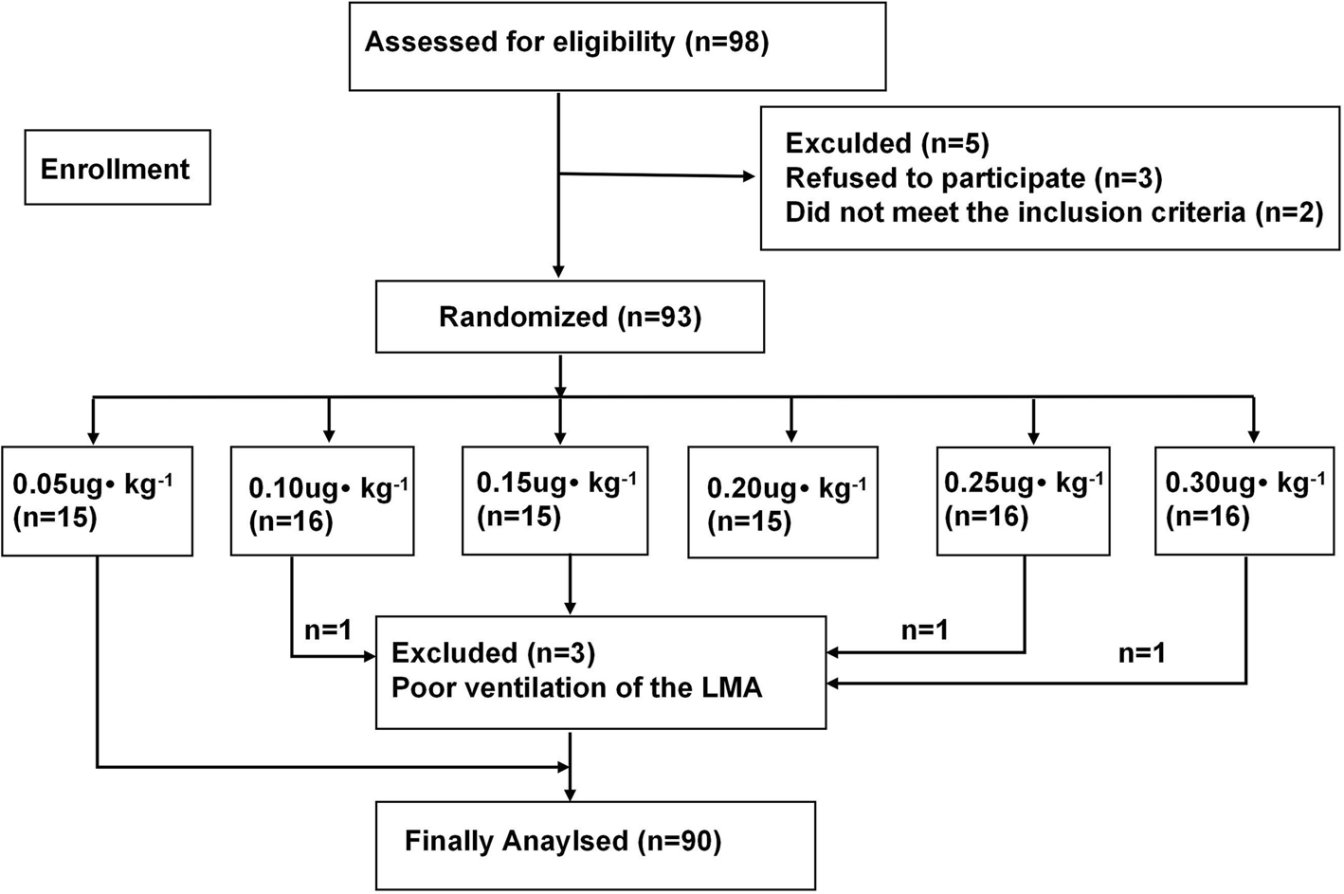
Participant cohort flow diagram

Ninety-eight patients were initially enrolled in this study, but three patients did not consent to participate, two patients were excluded from this study, and three patients were excluded after LMA insertion because of poor ventilation of the LMA. A patient cohort flow diagram is provided in Fig. 1.

**Fig. 2:**
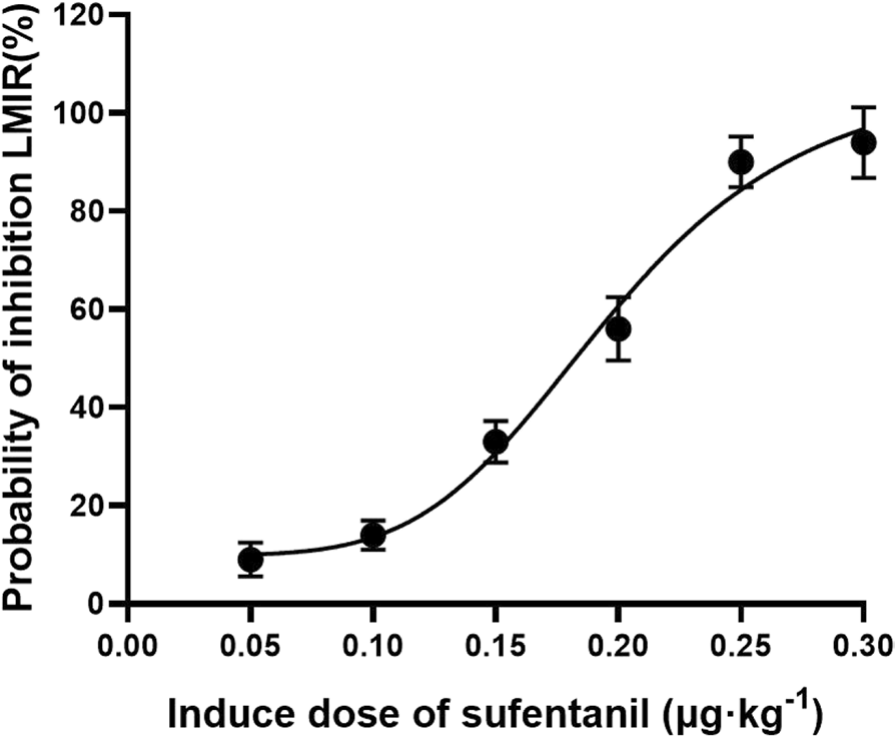
Dose–response curve of sufentanil for inhibiting the LMA insertion response. Probability unit vs. dose. Probit regression analysis of the dose–response curve revealed that the ED50 of sufentanil for the inhibition of the response induced by LMA insertion in geriatric patients was 0.18 μg kg^−1^ (95% CI: 0.16–0.21 μg kg^−1^), and the ED95 was 0.31 μg kg^−1^ (95% CI: 0.27–0.38 μg kg^−1^), and the probit(p) = -2.34 + 12.90 × ln(Dose)($\chi^{2}$ = 0.725, *p* = 0.948)

A dose–response curve was plotted by probit analysis and is shown in Fig. 2.

**Fig. 3:**
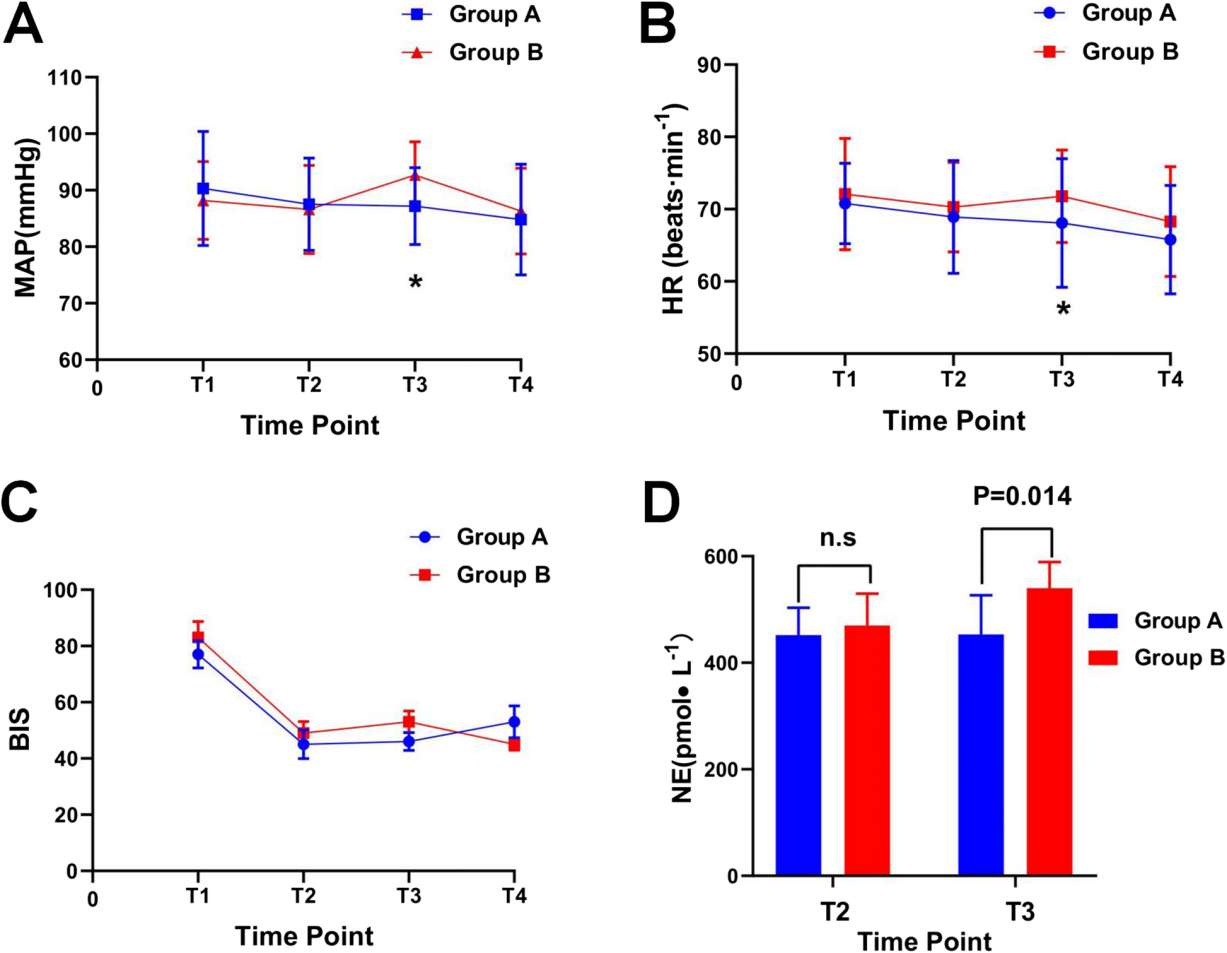
Comparison of MAP, HR,BIS,NE in both groups mean arterial pressure (**A**), the heart rate (**B**), bispectralindex (**C**), the plasma norepinephrine concentration(D):T1, 1 min before induction (T1);T2, 1 min after induction;T3, 1 min after LMA insertion;T4, 5 min after LMA insertion;*P < 0.05 for independent sample t-test comparing variales between two groups

The changes in MAP, HR, BIS, and the concentration of NE are shown in Fig. 3.

The MAP and HR of group A did not change significantly at any time point, but the MAP and HR of group B increased significantly at time point T3 and was significantly higher than that of group A (*P* < 0.001, *P* = 0.039; Fig. 3A and B). Norepinephrine did not differ significantly between T2 and T3 in group A after LMA insertion, whereas it increased significantly in group B at time point T3 and was significantly higher in group B than in group A (*P* = 0.014, as shown in Fig. 3D). The BIS decreased after induction. At the time of LMA insertion and 5 min after, the mean of the two groups remained between 40 and 60. There was no significant difference between the groups at each time, as shown in Fig. 3C.

The main adverse reactions in two groups are shown in Table 3.

**Table 3:** The incidence of main adverse reactions

	Group A *(n* = 57)	Group B (*n* = 33)	*P*-value
Respiratory depression	31 (54.4%)	7 (21.2%)	0.004
Bucking	6 (10.5%)	1 (3%)	0.416
Sore throat	3 (5.5%)	4 (12.5%)	0.415

Data are expressed as the number of cases (percentage), *P*-value: Chi-square test

Respiratory depression defined as the patient has irregular breathing rhythm and slow breathing rate, or SpO2 < 92%

In both groups, the MAP decline did not reach 20% before induction, the HR was not lower than 50 beats min^−1^, and there was no need to use ephedrine or atropine. The number of patients with apnea duration > 2 min was significantly higher in group A than in group B (*P* = 0.004). The incidence of choking in group A was 10.5%, higher than that in group B (3%), but there was no statistical difference between the two groups. The incidence of throat discomfort in group B was 12.5%, higher than that in group A, but there was no statistical difference between the two groups. Muscle stiffness, bradycardia, injection pain, muscle fibrillation, or intraoperative awareness did not appear in either group during the observation period.

## Discussion

The present study was to determine the ED50 of sufentanil for inhibiting the LMA insertion response in geriatric patients, the probit analysis shown that the ED50 and ED95 of sufentanil were determined based on whether effective LMA insertion was achieved, and they were 0.18 μg kg^−1^ (95% CI: 0.16–0.21 μg kg^−1^), and the ED95 was 0.31 μg kg^−1^ (95% CI: 0.27–0.38 μg kg^−1^), respectively.

The 3-point, 6-category scale used in the assessment of LMA insertion is a widely accepted and mature assessment method [13, 14]. A comprehensive assessment of LMA insertion is carried out through two indicators of LMA insertion difficulty (mouth opening and insertion resistance) and four indicators of insertion response (swallowing, choking, head or body movement, and laryngospasm). Effective LMA insertion is defined as a score of ≥ 16. The score scale was used to grade insertion conditions and that had been used successfully in previous studies [15–17], we adopted the scale method to grade insertion conditions because it is a classic and reliable evaluation scale.

In the study by Li on the optimal dose of sufentanil in Chinese pediatric LMA insertion [12], the ED50 and ED95 of sufentanil for LMA insertion were found to be 0.064 μg kg^−1^ and 0.177 μg kg^−1^, respectively. Choi et al. [18] showed that sufentanil can effectively reduce the cardiovascular response during double-lumen bronchial intubation under laryngoscopy, and 0.3 μg kg^−1^ can attenuate the cardiovascular response during double-lumen bronchial intubation. In a previous experiment, laryngeal masks were inserted in four consecutive cases without using sufentanil, the score of all patients was < 16 points on the 3-point, 6-category scale, and the hemodynamic changes of the patients were large. Based on this, we set sufentanil to six doses, namely, 0.05, 0.1, 0.15, 0.2, 0.25, and 0.3 μg kg^−1^.

Sufentanil is an opioid with a strong analgesic effect, which can effectively inhibit the stimulation of LMA insertion and maintain the stability of hemodynamics [19]. The MAP and HR of group A did not change significantly at any time point during the induction period. In group B, the MAP and HR increased significantly. At time point T3, MAP and HR were higher in group B than in group A (*P* < 0.001, *P* = 0.039). During this induction process, none of the MAP drops reached the 20% pre-induction level, and the HR did not fall below 50 beats min^−1^, so there was no need to use ephedrine or atropine.

The depth of sedation in patients with BIS reaction is mainly related to the dosage of diazepam, propofol, and etomidate [20]. In our experiment, 0.3 mg kg^−1^ etomidate was used. BIS remained between 40–60 for 5 min after LMA insertion, which fully met the requirements of anesthesia and sedation. In addition, there was no intraoperative awareness after surgery, which indirectly supports the idea that the depth of sedation in patients with BIS reaction is mainly related to the dosage of diazepam, propofol, and etomidate. It has been reported that sufentanil has a sedative effect and would affect the BIS value [21], but we found that there was no statistical difference in the BIS between the two groups at any time point; this discrepancy may be related to the small sample size in our study.

External stimuli can excite the body’s sympathetic nerves, causing the secretion of catecholamines in the body, resulting in increases in plasma dopamine, epinephrine, and norepinephrine [22, 23]. We found that during LMA insertion, norepinephrine was significantly increased in group B. At T3, the comparison between group B and group A was *P* = 0.014, which may be related to the stimulation of sympathetic nerves induced by LMA insertion. In group A, there was no significant difference in norepinephrine between two time points, indicating that sufentanil can effectively inhibit the stress response [24].

In the clinical application of sufentanil, adverse reactions sometimes occur, and the adverse reactions are often related to the dose used. The most common reactions are choking [25, 26] and respiratory depression [27], and the incidence of choking has been reported to be 31%–45.8%. In our study, the incidences of choking in groups A and B were 10.5% and 3%, lower than those reported, which may be related to the smaller dose we used and the slower bolus injection rate. After a patient is given sufentanil, the respiratory rate is often slowed down. Apnea usually occurs after etomidate injection, and the LMA is inserted 2 min later. If breathing does not resume, assisted or controlled breathing will be performed. The incidence of pulse oxygen below 92% in the whole process of induction is low, which ensures the safety of geriatric patients. Therefore, only the number of cases with apnea duration > 2 min was counted. The number of cases with apnea duration > 2 min in group A was greater than in group B, suggesting suggesting that respiratory depression might be related to the dose of sufenta nil. The ventilation difficulties caused by sufentanil mainly occur in the closure of the glottis or supraglottis [28], which can be quickly resolved after LMA insertion. In our study, machine-assisted breathing was safely performed after LMA insertion, and there was no difficulty in ventilation, so it is completely acceptable for geriatric patients to experience respiratory depression during sufentanil induction.

Some patients undergoing general anesthesia experience throat discomfort after LMA insertion, which is related to factors such as the poor positioning of the LMA and the repeated insertion of the LMA. The incidence rate is 9%–17.5% [29, 30]. In our study, the incidence of throat discomfort in the two groups was low, and there was no significant difference between the two groups. This may be because (1) we applied dyclonine mucilage containing local anesthetic ingredients on the back of the LMA [31], which reduced the patient's feeling of discomfort, and (2) after two unsuccessful attempts of LMA insertion, tracheal intubation was used decisivelyto avoid damage to the throat caused by repeated operations.

There were some limitations in the present study, the serum level of sufentanil was not measured, low concentrations of sufentanil affect postoperative pain scores, which increases the risk of postoperative delusions in elderly patients [32]. In addition, we used 0.05ug as the dose interval, which would require a small sample size and the statistical results would have increased bias,a smaller dose interval would provide a more precise result,which may provide more comprehensive data for sufentanil dosage during LMA insertion in geriatric patients. Moreover, we did not evaluate the muscle shivering response after etomidate injection, which might affect the assessment of insertion condition.

## Conclusions

Sufentanil can effectively inhibit the patient’s response to LMA insertion, with stable hemodynamics and small stress response. The ED50 and ED95 were 0.18 μg kg^−1^ (95% CI: 0.16–0.21 μg kg^−1^) and 0.31 μg kg^−1^ (95% CI: 0.27–0.38 μg kg^−1^), respectively.

## Data Availability

The datasets collected and/or analyzed during the current study are available from the corresponding author upon reasonable request.

## Abbreviations

LMA: Laryngeal mask airway
ED50: Median effective dose
ED95: 95% Effective dose
MAP: Mean arterial blood pressure
HR: Heart rate
BIS: Bispectral index
NE: Norepinephrine
CI: Confidence intervals
BMI: Body Mass Index
ASA: American Society of Anesthesiologists
